# The Impact of Regional COVID-19 Outbreak on Consumers’ Risk Perception of Purchasing Food Online

**DOI:** 10.3390/healthcare11111571

**Published:** 2023-05-26

**Authors:** Weijun Liu, Mengzhen Cao, Wojciech J. Florkowski

**Affiliations:** 1 College of Economics and Management, Shanghai Ocean University, 999 Huchenghuan Road, Shanghai 201306, China; 2 Shanghai Social Survey Center, Shanghai Ocean University Branch, 999 Huchenghuan Road, Shanghai 201306, China; 3 Department of Agricultural & Applied Economics, University of Georgia, 1109 Experiment Street, 212 Stuckey, Griffin, GA 30223-1797, USA

**Keywords:** COVID-19, regional effect, online food safety, consumer perception

## Abstract

This paper examines the perception of risk associated with the presence of coronavirus in food purchased online and online vs. offline food shopping during the COVID-19 epidemic. The influence of COVID-19 status on risk perception was tested using the data collected from 742 consumers between December 2021 and January 2022. The empirical approach distinguished between the epidemic’s status in a province (or region), city, and other areas of the country and applied the ordered logit technique. The regional and citywide epidemic increased the perception that online purchases carry the virus and are riskier than those made offline. Further examination showed that the regional/provincial epidemic created the perception that packaging or social media use were risk factors when purchasing food online. Heterogeneity analysis showed that risk perception was significantly higher in affected cities than in non-affected provinces or other provinces. Risk perception differed across five online food categories, with the highest levels for online-ordered meals and fresh products. Strengthening COVID-19 prevention and control in cities and the province, managing risk due to the handling of food purchased online, and government monitoring of social media use will lessen consumers’ risk perceptions and encourage the use of online food offers during epidemics.

## 1. Introduction

Between 2019 and 2021, the average annual growth rate of online catering service revenues exceeded 15%, a higher rate than for the catering industry in general [1]. Online food purchases have become important to national food security with the onset of COVID-19. Consumers across the globe have adapted the way they shop to changing conditions [2,3]. In the first half of 2022, China’s online food retail sales increased by 15.7% year over year [4]. A key attribute of food is that it is safe to eat [5]. China’s food safety has been steadily improving since the promulgation and implementation of the Food Safety Law in 2009. The “Global Food Safety Index” listed China’s (GFSI) score at 71.0 in 2019, or 35th among 113 participating countries, an improvement since 2017, when the score was 63.7, ranking China 42nd [6]. The coronavirus outbreak affected both local and global food safety systems [7]. There have been reports that frozen cod packaging was contaminated with the coronavirus, leading to possible transmission to consumers [8]. Coronavirus infections happen among food handlers who can transfer the virus to products at harvest, packaging, distribution, or marketing [9]. In terms of online food sales, consumers have become concerned about infection when buying food online [10]. The fear of infection causes less frequent or even termination of online food purchases [11]. The negative impact of COVID-19 on the safety of online food purchases calls for policy solutions. The safety risks of online food purchases considered in the current study focus on the possibility of a coronavirus infecting the consumer through the packaging of food ordered online. However, excessive consumer concerns could lead to unnecessary protective measures [12]. Due to the differences in epidemic prevention and control practices in different regions of China, the public’s ability to recognize hazards has been skewed. The risk of epidemics has been a widespread concern in the academic community, but there are few studies on the impact of COVID-19 on the risk perception of online-purchased food safety at the regional level. Although the risk perception of online-purchased food safety has been researched prior to COVID-19, there are few such studies in the context of the current epidemic. Therefore, this study fills a gap in the literature by identifying the drivers of consumer risk perceptions of the safety of food purchased online. Knowledge from the current study helps shape regional outbreak response policies and allows their effective implementation to improve consumer safety while sustaining online food sales during possible future epidemics.

The coronavirus epidemic has shown typical regional characteristics. The gradual spread of the virus was mainly from large urban centers and gateway cities to lower-level node cities in China [13]. The public’s perception of the epidemic risk varied across regions as the severity of the epidemic varied [14]. Regional differences led to a shift in prevention and control policies from “urban self-coordination” to “regional linkage” [15]. The prevention potential of a region may be strengthened through standardized and institutionalized inter-regional cooperation, reducing public risk perceptions of the epidemic [16]. Existing studies largely measure the epidemic’s impact using the number of confirmed cases and lockdown duration but lack regional focus. This paper attempts to scrutinize the impact of the pandemic on the risk perception of online-purchased food from a regional perspective using consumer survey data. Additionally, the role of social media in shaping perceptions was investigated because the limitations of direct personal interaction were replaced with searching for information about the pandemic and food purchase options on electronic platforms. Observing China is insightful because it has a large population, a large area, and varying regions. The current study selects City C of Province P (hereinafter referred to as C city and P province) as the regional COVID-19 entry point. The remaining country’s area is under control. The details of such distinction are provided in the subsequent sections.

The contributions of this paper to the existing literature are to: (1) deepen understanding of risk perceptions of online food purchases caused by coronavirus during regional public health emergencies; and (2) ascertain ways to reduce the effect of pandemics on safety perceptions of online-purchased food. The knowledge gained will help to shape policies and public education programs to assure consumer safety perceptions when relying on online food purchases during public health emergencies, including planning for future pandemics.

## 2. Literature Review and Research Hypotheses

The 21st century epidemics prior to COVID-19 included severe acute respiratory syndrome (SARS), influenza A H1N1 (swine flu), and Middle East respiratory syndrome (MERS) [17]. However, none of the earlier epidemics affected such a large number of people or were associated with lockdowns characterized by impinging on individual mobility and triggering anxiety. The feeling of being threatened by an unknown virus motivated consumers to protect themselves, a choice consistent with the protection motivation theory [18]. Generally, food safety risk perception refers to consumer judgment [19]. Consumer judgment is shaped by governmental actions to contain an outbreak and media communication [20], including social media platforms. The appraisal of an individual ability to cope with the threat in the current study is based on consumer proximity to the area affected by the pandemic. The approach is consistent with the temporal construal theory that the proximity to the event in time explains how consumers differ in perceiving the same information [21]. The implied measure of proximity in this study is the spatially varying number of confirmed COVID-19 cases, as described in the subsequent text.

### 2.1. Online Food Safety Risk Perception

A two-factor model and a multidimensional scale have been used to measure consumer perceptions of food safety risk. Gao et al. (2020) [10] showed that consumers worry about whether personnel and couriers who handle and deliver foods are infected with the virus. Xu et al. (2021) [22] report that the perception of higher infection risks from online shopping than offline shopping inhibits consumers’ migration to online purchases. During the pandemic, the number of online food orders increased, but the environmental safety, delivery quality, and hygiene of food packaging were questioned [23].

The existing research on the risk perception of online food purchased during COVID-19 presents two views. First, the risk of online food containing the coronavirus is low for two reasons. In the early stages of the COVID-19 outbreak, the virus may have been present in other purchasing environments compared with offline purchasing [2]. The supply chain of online purchases is shorter, which limits food exposure to the possibility of becoming infected with the coronavirus and, therefore, makes it less likely to carry the virus [3]. In the later stages of the pandemic outbreak, online express delivery adopted non-touch distribution and strengthened disinfection measures at all stages [24]. The probability of consumers becoming infected by the received goods has likely been reduced compared with the initial stage [25]. Therefore, it appears that consumer risk perception is a lesser barrier to online food purchases [26].

The opposite view is that the risk of eating online food containing coronavirus remains high because COVID-19 has a strong mutation ability and a high transmission rate [27,28]. The survivability of the virus during transportation at a temperature of −18 °C could exceed 21 days [29]. Despite the implementation of various control measures (for example, non-touch and alcohol disinfection), the virus may still be present on the surface of food or packaging [30]. When consumers buy food online, they may still become infected after receiving, unpacking, or preparing the food [10]. Food can carry the virus indirectly, and an indirect pathway of transmission needs to be recognized in developing prevention measures [31]. Among food groups, frozen food packaging has been identified as a source of COVID-19 infection [32]. Concerned about their own protection, consumers may reduce the purchase of online food [11] and apply unnecessary disinfection practices to packaging and food purchased online (e.g., washing food with soap and non-food-grade chlorine bleach) [33].

### 2.2. Regional COVID-19 Epidemic and Risk Perception of Online-Purchased Food Containing Coronavirus

Joint regional pandemic prevention and control regimes continually evolve, and their effectiveness still needs to be studied in light of the dynamic epidemic situation. Ali and Keil (2006) [34] report that transportation and cross-regional economic and social connections inevitably allow an epidemic to rapidly spread across regions and countries. Other studies have found that population flow does not necessarily lead to the virus spreading but largely depends on the intensity of regional joint prevention and control [35]. Refining regional prevention and control measures reduces the spread of the epidemic and avoids the risk of large-scale outbreaks. Therefore, this paper examines the impact of the epidemic from the perspective of joint regional prevention and control.

Studies of the COVID-19 pandemic focusing on the risk perception and safety of online-purchased food are limited. The existing studies have focused on three aspects of COVID-19, among them the severity of the pandemic. Liu Lingzhi (2021) [36] classified the effects of the epidemic as severe or mild. Severe levels involve more than ten thousand cumulatively confirmed cases in a province. A mild level implies between one thousand and ten thousand cumulative confirmed cases in a province. Similarly, Meng Xiangwei (2022) [37] classified the impact into severe, sub-severe, intermediate, and light epidemic levels according to the confirmed cases. The results showed that as the severity of the pandemic increased, the public’s perception of infection increased. Other studies looked at the number of confirmed COVID-19 cases. A higher number of confirmed cases increases risk perception [30]. The third aspect of research is lockdown duration and severity. The latest research found that the length of lockdown increased the perceived risk of becoming infected with COVID-19 [38]. Habib and Hamadneh (2021) [26] proposed that the COVID-19 pandemic increased risk perception and had consequences for online food shopping.

However, there is a lack of empirical studies considering a regional dimension. Currently, the epidemic in many areas of China is small-scale and involves sporadic regional characteristics. The practical significance of such regional variation needs more research from the perspective of prevention and control measures coordinated among regions. To address the regional variation, this paper proposes the following hypothesis:

**Hypothesis** **1** **(H1).** *Regional COVID-19 severity increases consumer risk perception of becoming infected by online-purchased food.*

### 2.3. Online Food Purchase as Infection Source

The COVID-19 pandemic has exposed the fragility of the fresh food supply chain, exacerbating information asymmetry and affecting consumer perceptions of the safety risks of online-purchased food. Production, processing, logistics, and sales are viewed as hazards because they can facilitate infecting consumers [39]. The epidemic prevention and control measures extend the delivery time, creating the bullwhip effect in the supply chain and blocking information flow [40], exacerbating information asymmetry. The position of consumers as information recipients from other supply chain actors leaves them unable to protect their own interests and strengthens their perception of virus dissemination risk. This study proposes a second hypothesis:

**Hypothesis** **2** **(H2).** *Regional COVID-19 severity indirectly influences consumer risk perception of online-purchased food as a source of infection.*

### 2.4. Social Media Effect

The accuracy of social media may be difficult to assess. Social media operators may spend a limited amount of resources on content verification. In the face of a large amount of information, the audience’s ability to discriminate among multiple messages is uneven, and inaccurate information could influence consumer behavior. The outbreak of COVID-19 brought uncertainty. Consumers, regardless of the severity of the lockdown, searched for epidemic-related information using social media platforms. The opinions expressed through social media might have contributed to anxiety, strengthened risk perceptions of the pandemic [41], and indirectly affected the perception of the safety of online-purchased food. Netizens (i.e., participants in social media platforms) engaged in online discussions about food safety and influenced the opinions and behavior of others, creating a herding effect [42] and spreading perceptions of food safety hazards associated with online purchases. The COVID-19 pandemic revealed the influence of social media platforms as a source of information relevant to consumer food purchase behavior. Based on the above discussion, this paper proposes a third hypothesis:

**Hypothesis** **3** **(H3).** *The regional impact of COVID-19 enhances consumer risk perception of online-purchased food containing coronavirus through social media use.*

## 3. Materials

### 3.1. Survey Design and Data Collection

**Figure 1:**
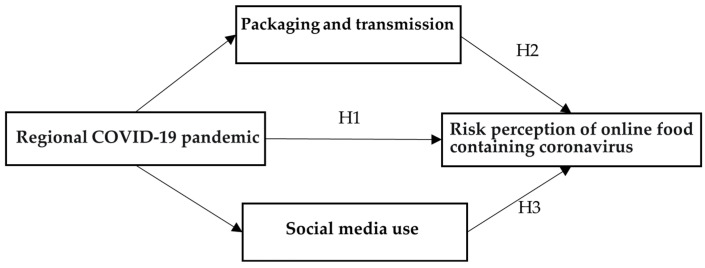
The regional COVID-19 pandemic effect on consumer risk perception of online food containing coronavirus.

The focus on regional COVID-19 impact guided the construction of the questionnaire and included the design, a pilot test, and questionnaire revisions following the pilot. The final questionnaire consisted of three parts, corresponding to Figure 1. General questions were asked about regional COVID-19 pandemic features. A separate set of questions probed respondents perceptions of the risk of virus transmission through the packaging of online-purchased food and the use of social media. Respondents were also asked to share information about their socio-demographic characteristics and income. Ultimately, 13 variables were specified using the responses obtained to implement this study. The questionnaire is included in Appendix A.

The survey was conducted between December 2021 and January 2022. Following the National Health Commission of China’s report that the vast majority of confirmed cases at that time (December 2021–January 2022) were in Xi’an, Shaanxi Province, that province is defined as the epidemic province, or P province, and Xi’an City is defined as the epidemic city, or C city. At the time, there were few confirmed cases in other regions or provinces, which served as a control. The confirmed COVID-19 cases from December 2021 to January 2022 can be found at: http://www.nhc.gov.cn/xcs/yqtb/list_gzbd_15.shtml (accessed on 25 August 2022).

The questionnaire was distributed through online network channels in two stages between January 12 and January 22, 2022. In the first stage, the research team distributed the questionnaires through WeChat and a mobile phone network. To encourage participation in the survey, a respondent was offered a gift once the questionnaire was completed and met the quality screening. A total of 216 valid questionnaires were collected, including eight in C cities in P province, four in non-C cities in P province, and 204 in other regions. In the second stage of the survey, 526 valid questionnaires were collected with the help of professional survey companies, including 113 in the C city of P province, 103 in non-C cities of P province, and 310 in other regions. In both stages, a total of 121 questionnaires were collected from the C city of P province, 107 from non-C cities of P province, and 514 from other regions, for a total of 742 questionnaires. Table 1 details the number of collected questionnaires.

### 3.2. Sample Description and Variable Selection

Table 2 shows selected descriptive statistics and variable definitions. The sample consists of answers provided by 742 respondents. The first dependent variable to test the importance of a regional effect is the consumer perception that COVID-19 is contracted by food purchased online. The respondents expressed their risk perception by choosing an option along the five-step scale (see Table 2 for details). The second relationship specified the dependent variable, placing the online food purchase in a specific context. Following Zanetta (2021) [19], the current study concerns the consumer’s higher risk perception of COVID-19 spread by food purchased online as compared to off-line food purchases. The respondent chose an option along the five-step scale where the choice was ordinal and implied a higher risk perception along the scale. The ordinal nature of the response determines the estimation technique and suggests the use of the ordered logit.

Two explanatory variables capture consumer perceptions. Specifically, the perception that infection is spread by viruses present on packaging or food purchased online and the use of social media to learn about COVID-19. Social media postings include unverified information, which, in the case of searching for information about COVID-19, is of importance to disoriented consumers. Survey respondents indicated their perceptions by selecting one option along the five-step scale (Table 2).

The sample includes slightly more females (54.31%) than males. The majority of respondents were 23–40 years of age (67.12%). Most respondents received university education corresponding to a bachelor’s degree (58.63%). The monthly after-tax income, measured by seven categories, is relatively high and exceeds 6000 yuan (mean 4.14), but the large standard deviation (1.63) suggests wide dispersion. The majority of respondents reported a family size of three (44.2%). Among the vulnerable respondents were a relatively small number of pregnant women (1.35%), but the share of elderly people was substantial (24.80%). Overall, the sample includes relatively young individuals, well-educated respondents, and relatively high-income respondents, all features common to large agglomeration populations.

The respondent’s personal characteristics (gender, age, education, and income), family demographic characteristics, and the identification of vulnerable groups (pregnant women, the elderly) are control variables in the study (Table 3). The explanatory variables are classified as key explanatory variables, variables accounting for the mechanism of perceived virus spread, and control variables (Table 2). The key variables include the regional effect and the city effect, reflecting the regional epidemiological conditions due to COVID-19.

## 4. Methods

The ordinal nature of the dependent variables suggests an ordered logit as the estimation technique. The technique is applied in the estimation of two empirical equations modeling the effect of the regional COVID-19 epidemic on the perception of risk because of the purchase of food online and the perception that online food purchases represent a higher risk of becoming infected than buying food offline at conventional retail outlets. Empirical analysis is performed using Stata software (SE 16.1 version, College Station, TX, USA).

Next, the study employs a mediation effect model to test the effect of two variables of interest: the transmission of the virus by the packaging of food purchased online and the use of social media by the respondent to seek COVID-19 relevant information. To test the mediation effect, the study uses the Karlson, Holm, and Breen (KHB) method. The method permits the calculation of the total effect, direct effect, and indirect effect using the logit or probit technique. The KHB model can decompose the total effect into direct and indirect effects by comparing coefficients in the model [43]. KHB is compatible with various standard Stata estimation commands. The KHB method ensures that the measured coefficient is on the same scale and is not affected by scaling to identify problems.

To assure the robustness of the estimated equations modeling the perceptions of the risk of becoming infected by a virus present on the packaging of food bought online and the online purchase being riskier than buying offline, both equations were subjected to a test. The test involved two regression techniques to verify the directional effects and statistical significance of coefficients. The baseline model is robust, proving that the region and city affected by the epidemic increase consumer perceptions of safety hazards associated with online food purchases.

The heterogeneity analysis further identified the effects of selected explanatory variables on consumer perceptions of hazards involving the purchase of food online. In particular, the analysis distinguished between the influence of the location, i.e., residing in a non-affected region, an affected province, or an affected city. Additionally, the heterogeneity analysis discerned the effects with regard to five food groups purchased online, namely aquatic products, fresh agricultural products, fruits and vegetables, online-ordered meals, and shelf-stable foods.

## 5. Empirical Results

### 5.1. Baseline Regression

The empirical specification is as follows:

(1a) $$Y_{1}=\alpha_{1}+\beta_{1}COVIDR_{i}+\gamma_{1}Packaging_{i}+\delta_{1}Social\;media\;use_{i}+\varepsilon_{1}Z_{i}+\theta_{i1},\left(i=1,2,\ldots ,n\right)$$

(1b) $$Y_{2}=\alpha_{1}+\beta_{1}COVIDC_{i}+\gamma_{1}Packaging_{i}+\delta_{1}Social\;media\;use_{i}+\varepsilon_{1}Z_{i}+\theta_{i1},\left(i=1,2,\ldots ,n\right)$$

In the empirical relationship, $\;Y_{1}$ is the dependent variable is consumer perception of the risk of COVID-19 infection from online-purchased food. Equation (1a) shows the relationship only for investigating the role of the regional epidemic, but another relationship is specified where the regional effect is replaced by the city epidemic situation. $Y_{2}$ is the perception of higher risk from online than offline food purchases (1b) and is specified in two versions, accounting for the regional ($COVIDR_{i}$) and city effects ($COVIDC_{i}$) separately. There are four equations in total, two emphasizing the regional epidemic importance (province P), two emphasizing the city epidemic (city C), and two different dependent variables of risk perception associated with food purchased online (see Table 2 for details). $COVIDR_{i}$ is the regional epidemic in Province P while $COVIDC_{i}$ is the epidemic in City C, $Packaging_{i}$ is the perception of virus transmission through the packaging or food in AN online purchase. $Media_{i}$ is respondent reported information search about COVID-19 on social media platforms, $Z_{i}$ includes the socio-demographic attributes of the respondent and income treated as control variables (Table 2), $\theta_{i}$ is the random disturbance term, and α, β, γ, δ, and ε are parameters to be estimated.

Table 4 shows the estimation results of two relationships regarding factors influencing risk perception of the presence of COVID-19 on food purchased online, distinguished by the effect of the regional epidemic in Province P (column (1)) or the epidemic in City C (column (2)). Similarly, columns (3) and (4) show whether the regional or city epidemic influences consumer perceptions of online food purchases as posing a higher risk of infection than offline purchases. The regional/province epidemic situation increases the risk perception of consumers regarding online-purchased food as a source of coronavirus, and it also increases the perception that online food purchase is riskier than offline purchase (Table 4). The effect of the city epidemic situation has a similarly increasing influence on consumer risk perception regarding coronavirus presence in food purchased online or that online purchases are riskier than offline food purchases (Table 4). These results indicate that consumer risk perceptions of online food safety increase as the respondent’s location changes from a non-P province to a C city or when the respondent is a city resident. The effect is also positive if the city was affected by the epidemic. Overall, the closer the consumer resides to the epidemic area, the higher the consumer’s perception of online-purchased food as posing an infection risk, a result consistent with Hypothesis 1.

### 5.2. Mechanism Analysis

Further examination of whether a regional/province epidemic can indirectly affect consumer risk perception of viruses present on online-purchased food packaging or in its contents, as well as any social media use, is based on the KHB decomposition (Karlson 2012) [44]. The mediation effect model proposed by Baron and Kenny (1986) has been applied when the explanatory variable is continuous. In the current study, both explanatory variables are discrete and ordered [45]. Such decomposition applies to cases of the discrete explanatory variable [46].

As for the mediation effect of the COVID-19 infection from online-purchased food, testing involves the following steps (in the case of tracking the influence of a regional/province epidemic):

(2) $$Y_{i}=\alpha_{2}+\beta_{2}COVIDR_{i}+\lambda_{2}Z_{i}+\varepsilon_{i},\left(i=1,2,\ldots ,n\right)$$

(3) $${\mathrm{Packaging}}_{i}=\alpha_{3}+\beta_{3}COVIDR_{i}+\lambda_{3}{Ζ}_{i}+\varepsilon_{i},\left(i=1,2,\ldots ,n\right)$$

(4) $$Y_{i}=\alpha_{4}+\beta_{4}COVIDR_{i}+\gamma_{4}{\mathrm{Packaging}}_{i}+\lambda_{4}{Ζ}_{i}+\varepsilon_{i},\left(i=1,2,\ldots ,n\right)$$

where $Y_{i}$ is consumer risk perception of the presence of coronavirus on food purchased online; $COVIDR_{i}$ is the key variable (measured by two variables, *Region R* and *City C*—see Table 2); $Chain_{i}$ is one of the mechanism variables, $Z_{i}$ includes socio-demographic attributes and income of the respondent, $\varepsilon_{i}$ is the random error term, and α, β, γ  and λ are parameters to be estimated.

Equation (2) shows the regional COVID-19 direct influence on consumer risk perceptions of food purchased online. Equation (3) show the regional COVID-19 influence on the risk perception of the coronavirus presence on packaging or food purchased online. Equation (4) show the total effect of regional COVID-19 and the perceived risk of the coronavirus’s presence on the packaging of food purchased online on the dependent variable. Similarly, the mediation effect equation examines the influence of social media use.

The indirect effects of virus infection from the packaging of food purchased online are statistically significant (Table 5). The positive effect of the epidemic in regions/province, and cities increased consumer perception of the compromised safety of food purchased online. The specific indirect effect of COVID-19 present on the online-purchased food was 15.84% (percentage of indirect = Indirect effect/Total effect x 100%, or 15.84% = 0.071/0.448*100%). Similarly, the indirect effect of the epidemic in regions/province increased consumer perception that online purchases were riskier than offline purchases by 19.25%. In the case of the effect of an epidemic in the city, indirect effects amounted to 19.51% and 16.42%, respectively.

Several reports indicated that the surface of frozen food packaging contained virus from virus-infected handlers, causing the spread of the epidemic [47,48]. Consumers receiving the online-ordered food are the last link in the supply chain and dependent on the actions of others earlier in the chain. Consumers are uncertain of the hygiene at different stages of the supply chain. The information asymmetry within the supply chain makes consumers uneasy about online food safety. Analysis shows that the potential presence of coronavirus on online-purchased food packaging is one way a regional/province epidemic influences consumer risk perception. Hypothesis 2 has been supported.

The indirect effect of social media use is statistically significant and positive in two equations testing the effect of the regional/province epidemic (Table 6). The indirect effect of social media use accounted for 4.77% (column (1)) and 7.06% (column (2)) in both equations associated with the regional/province epidemic. Columns (3) and (4) show the indirect effect of social media use, accounting for 4.66% and 4.39%, respectively, in both equations associated with the city epidemic. The indirect effect of social media use is significant and indicates that regional epidemics prompt consumers to seek information on social media platforms. The more consumers rely on social media to obtain epidemic-related information, the higher the perceived risk of online-purchased food.

The pandemic reduces consumers’ sense of security and trust in online-purchased food safety. The closer consumers are to the pandemic-affected region/province or city, the more frequently they search to obtain information through convenient, accessible social media, likely to alleviate the fear of the unknown heightened by the information asymmetry. The multi-modal presentations on social media platforms [49] that are visually and audibly stimulating, the feature that “everyone has a microphone”, and the possibility that information is untrue pose a monitoring challenge and could mislead the social media audience. The results show that social media use is a path for regional/province epidemics to affect consumer perceptions of the safety of online food purchases, supporting Hypothesis 3.

In summary, the regional/province epidemic effect not only directly influences consumer perceptions of the safety of online-purchased food but also indirectly affects consumer perceptions through the effect of social media use. The indirect effect of the presence of coronavirus on online-purchased food is greater. Both hypotheses 2 and 3 have been supported.

### 5.3. Robustness Test

The robustness of the empirical results is checked using the replacement model and replacement key variables. The ordered probit and OLS regression models were estimated using the same sample (Table 7). The use of different estimation techniques to examine the consistency of the result is used for robustness checks. The perspective on robustness tests varies for different research purposes. Although the dependent variable in the current study is an ordered discrete variable, the risk perception of online food safety can also be regarded as a continuous variable, and the OLS model can be used [50]. Results in Table 7 show the reasonable fit of the two relationships estimated using two techniques. The OLS regression directional effects and significance were the same as those obtained using the ordered logit. The baseline model is robust, proving that the region and city affected by the epidemic increase consumer perceptions of safety hazards associated with online food purchases.

### 5.4. Heterogeneity Analysis

Regional factors associated with the epidemic significantly influence consumer perceptions of the safety of online food purchases. The earlier results show only the average effect using the whole sample and do not consider the heterogeneity of the effect of regional epidemics on consumer risk perception associated with online food purchases. A heterogeneity analysis accounted for regional, city, and food type effects. The estimation results are shown in Table 8 and Table 9. This paper also examines the heterogeneity of gender, education, and the sensitivity of consumer perceptions of the safety of online food purchases by category. Due to space limitations, the specific results are not discussed in this paper. Full results are available upon request.

#### 5.4.1. Regional/Province and City Influences

The results indicate that consumers in non-affected cities in the epidemic-affected province, compared to non-affected provinces, have significantly higher risk perceptions of coronavirus on online-purchased food (Table 8). The results show that consumers in the epidemic-affected city have a higher perception of the risk of virus presence on the packaging of online-purchased food and that online purchases are riskier than offline food buying compared to consumers in the epidemic region/province and non-affected cities. The closer a respondent resides to the epidemic city, the more concerned they are about the safety of online food, compared to “non-P province”. The coefficients of “Province, but not C city” (0.616) and “C city” (0.652) are significant.

The results support the idea that central, provincial, and local governments should focus on prevention and control around the affected cities, especially in the neighboring cities within the province. Such efforts involve information sharing and strengthening public health monitoring, especially in high-risk occupations including catering and courier delivery. The Joint Prevention and Control Mechanism Comprehensive Team of the State Council formulated and issued the ninth edition of the Prevention and Control Plan for Novel Coronavirus Pneumonia, proposing to strengthen regional joint prevention and control on the basis of targeted prevention and control. See http://www.gov.cn/xinwen/2022-06/28/content_5698168.htm (accessed on 29 September 2022).

#### 5.4.2. Perceived Risk Differences in Various Food Categories Purchased Online

The five categories of food purchased online include: online-ordered meals, fresh agri-products, vegetables and fruits, frozen food, and shelf-stable food. Among them, fresh agri-products include fresh aquatic products, meat, poultry, eggs, and dairy products. Frozen food includes frozen aquatic products, seafood, meat and poultry, and quick-frozen flour food (dumplings, buns, etc.). Table 9 shows that among 742 respondents, over half “agreed” or “completely agreed” that frozen food (64.69%), fresh agri-products (63.21%), and vegetables and fruits (51.07%) may contain SARS-COV-2. The percentage of consumers who “agreed” and “completely agreed” that the online-ordered meal contained a virus was 45.15%. In the case of shelf-stable food, the percentage was relatively low (38.67%).

The marginal effects estimation results show (Table 10) that the regional COVID-19 epidemic increased by one unit, and the probability of consumer perception increased by 5.21% that fresh agri-products pose a very high risk because they carry the virus (significant at the 1% level). Agri-products are followed by frozen food (3.15%) and online-ordered meals (1.86%).

Consumers have the highest perceived risk of fresh agri-products carrying COVID due to the effects of the regional epidemic. The coronavirus can infect animals, and animal COVID-19 cases have been reported [51]. Consumers are concerned that the proximity to the affected areas poses a higher possibility of cross-infection between animals, aquatic products, meat, poultry, eggs, and dairy products.

Frozen food was the online food category that most consumers felt may contain SARS-COV-2 (Table 9). China CDC Weekly (2021) (CDC: Chinese Center for Disease Control and Prevention) cited a study by Bai et al. [47] that detected COVID-19 viral RNA on the surface of imported frozen seafood and meats and their packaging in 18 provincial divisions in China. The viral RNA must be viable to be infectious, but few consumers have the specific knowledge to discern the difference.

Online-ordered meals are the third category of food that consumers are worried may contain viruses. During the epidemic, online catering services supplemented the food distribution system, raising concerns about a higher risk of infection due to contact with high-risk groups such as meal delivery couriers.

Vegetables, fruits, and shelf-stable foods, commonly purchased online, are a daily source of nutrition, and the possibility of carrying a virus has caused less concern among consumers. Indeed, the case of transmission through those products has not been reported, supporting the absence of a perceived threat from those products to consumer safety.

## 6. Conclusions and Policy Implications

### 6.1. Conclusions

This study investigated the influence of province and city affected by the COVID-19 epidemics on risk perceptions that online-purchased food is a source of possible infection using survey data collected between December 2021 and March 2022. Online purchases offer uninterrupted access to food. Reducing perceptions of virus transmission using this distribution channel to limit the public’s anxiety while ensuring food access is therefore important. The results showed that the regional epidemic significantly increased consumer perceptions of the risk of food packaging carrying the virus that can infect the delivery recipient. In addition, the regional epidemic increased the perception that online food purchases are riskier than buying food offline. The use of social media is also linked to an increased perception of the risk of virus transmission through online food purchases. The robustness of the results was verified using additional testing.

The results of heterogeneity analysis showed that, compared with the provinces not affected by the epidemic, the province epidemic had the greatest influence on purchase risk perception of online food safety in the epidemic-affected cities, followed by the effect of non-affected cities. Since proximity to the area affected by the epidemic is a key factor related to the level of risk perception, efforts to inform the public about precautions taken in food distribution are important, and the dissemination of information pertaining to the safe handling of online-ordered food through social media is warranted. Online food ordering seems less likely to expose the recipient to the virus than shopping in conventional food retail outlets because of the reduced number of people a recipient meets.

In the current study, the effect of the epidemic had a particularly great impact on the risk perception of the safety of online aquatic products and online-ordered meals, suggesting that to assure the public of the safety of those products, special efforts regarding the handling of such foods (such as cooking methods that destroy the viability of any virus) have to be conveyed by restaurants offering meal delivery. The efforts of the meal preparer need to be supported by information from the government agencies responsible for monitoring the enforcement of food safety regulations, including the monitoring of regulations covering couriers delivering online-ordered meals. The diligence of such efforts is essential to building trust in product safety.

### 6.2. Policy Implications

The government has an opportunity to select special measures to further improve the online food supply safety system under the real conditions of epidemics, especially regional outbreaks. Unified and standardized prevention and control method development should allow for input from consumers regarding their views of food safety during the recent pandemic. Once developed, the prevention and control methods have to be communicated to the public and require the training of managers in charge of future outbreaks. It is essential to assure a degree of flexibility in prevention and control management that discriminates between the affected cities and adjacent cities, especially those not affected, in the same province and in neighboring provinces.

Social media can inform consumers of steps to take when handling deliveries to protect against the disease. An understanding of how to handle various types of food empowers the consumer and helps reduce the perceived risk of contracting the disease. Improving the transparency of the methods used in handling ordered food in the preparation of the shipment, tracking the shipment, and tracing the original suppliers will enhance the safety of food under normal operating conditions as well. Educating the public will assure that food safety is a priority. Logistics systems have to be upgraded, the speed of delivery must be increased, and safe distribution must be planned in advance to allow continuity of deliveries, according to the latest epidemic information. Sharing such information with the public will minimize information asymmetry in the online food supply. Strengthening the prevention of misinformation on online social media platforms by releasing information through official channels needs to be considered. Such efforts would empower consumers to understand the food safety risks associated with the epidemic and help temper the negative effects of an epidemic.

In terms of regional prevention and control strategies, it is stressed that all regions should have both uniform prevention and control systems that are distinct and specific to the conditions in the regions in which they are implemented. Uniformity of prevention and control implies that all regions can have access to records allowing for the tracing of food shipments and monitoring of the health of personnel handling food supplied to distributors and outgoing shipments. This allows public health managers to directly and quickly connect with others or share information when necessary, such as in the event of a regional outbreak. Regional prevention and control management emphasizes the differences in local levels of public emergency, geographical conditions, and local customs. Regions are encouraged to create unique and effective prevention and control management methods specific to their conditions. In terms of regional prevention and control priorities, joint prevention and control should be strengthened in urban border areas.

To minimize and possibly eliminate the risk of disease transmission by the packaging of online food deliveries of either raw food or ordered meals, it seems warranted to review the existing handling procedures and possibly formulate new working procedures. Emphasizing food traceability and regulation enforcement, for example, HACCP standards, can enhance food processing safety.

The role of social media has proven important in shaping consumer perceptions during the pandemic. Not surprisingly, restrictions prevented many essential aspects of daily living, including food shopping, and consumers came to rely upon available sources of information and communication enabled by social media. Social media postings frequently list erroneous or unverified information, adding to the anxiety caused by the fear of becoming infected with an unfamiliar virus. Operators of the social media platforms need to develop and apply methods and procedures for screening the posted information, especially as it pertains to food safety, distribution, and handling. Government regulation may foster digital innovation to improve the accuracy of postings with a view to managing future outbreaks more effectively. Users of social media need to be educated on how to verify information and accurately assess its credibility.

As the world gradually enters the post-pandemic era and countries relax public control programs or declare the end of the pandemic, one should bear in mind the impact of the pandemic on the safety of online-purchased food. The experience of the pandemic is invaluable in keeping online food safe and offers opportunities to undertake the challenge of minimizing the transmission of disease through packaging in online food purchasing.

### 6.3. Limitation and Future Work

The study was limited in the methods of data collection that could be employed. Under the restrictions imposed in the COVID-19-affected areas, the online completion of a questionnaire was the only viable method. Face-to-face interviews were not possible during that time. Consequently, those without broadband Internet or cell phone service, among them older consumers, might have been excluded from the study. Therefore, the survey sample may not fully reflect the population of consumers, although those without access to the Internet could not use the online food purchase opportunities. A future study that applies the conventional face-to-face interview method may establish if and to what extent the current sample missed potential respondents.

The focus of this study was on a single area affected by the COVID-19 pandemic. A future study needs to involve other regions in China or even other countries. Additionally, a larger regional sample could verify the statistical results obtained in this study and provide further insights. A future project may also provide perspectives on online food shopping by probing for risk perceptions after the COVID-19 pandemic, showing to what extent food choices and shopping habits may have shifted and how such changes need to be considered in managing future outbreaks.

## Figures and Tables

**Table 1:** Number of questionnaires collected from respondents residing in the city, province, and outside the province.

Sample Area	P Province, C City		P Province, not C City		Outside P Province		Total	
	Number	%	Number	%	Number	%	Number	%
First stage	8	1.08	4	0.54	204	27.49	216	29.11
Second stage	113	15.23	103	13.88	310	41.78	526	70.89
Total	121	16.31	107	14.42	514	69.27	742	100

**Table 2:** Variable definitions and descriptive statistics.

Variable	Variable Definition	Mean	Standard Deviation
**Dependent variable**			
Food purchased online source of COVID-19	“Degree of risk of COVID-19 from food purchased online ”: 1 = no risk at all; 2 = little risk; 3 = neutral; 4 = higher; 5 = very high risk	3.24	0.88
Online purchase higher COVID-19 risk	“Degree of agreement that online-purchased food is more susceptible to COVID-19 contamination than off-line food”: 1 = completely disagree; 2 = basically disagree; 3 = neutral; 4 = agree; 5 = totally agree	3.27	1.06
** Key explanatory variable **			
*COVIDR_i_*	Respondent location. 1 = other regions of non-P province; 2 = P province, but not C city; 3 = C city	1.47	0.76
*COVIDC*	1 = the city with the major epidemic (C city, P province); 0 = other	0.163	0.37
** Mechanism variables **			
Packaging	“Degree of agreement that online food ingredients or packaging surfaces may contain COVID-19/or its variants”: completely disagree = 1; basically disagree = 2; neutral = 3; agree = 4; total agree = 5	3.76	0.86
Social media use	“Do you use online social media to learn about COVID-19?”, yes = 1; no = 0	0.57	0.50
**Control variables**			
Gender	Male = 1; Female = 2	1.54	0.50
Age	Younger than 22 = 1; 23 to 30 = 2; 31–40 = 3; 41 to 50 = 4; older than 51 = 5	2.78	1.02
Education	1= less than high school; 2 = college; 3 = undergraduate; 4 = Master; 5 = Doctoral degree	3.11	0.89
Net monthly income	Less than 2000 yuan = 1; 2001–4000 yuan = 2; 4001–6000 yuan = 3; 6001–8000 yuan = 4; 8001–12,000 yuan = 5; 12,001–20,000 yuan = 6; more than 20,001 yuan = 7	4.14	1.63
Household size	1 person =1; 2 persons = 2; 3 persons = 3; 4 persons = 4; 5 or more = 5	3.31	1.05
Pregnant female in the family	1 = yes; 0 = no	0.01	0.16
Older than 60 years	1 = yes = 1; 0 = no	0.25	0.43

**Table 3:** Frequencies and percentage shares of the socio-demographic characteristics and income of respondents (n = 742).

Variable Name	Description	Frequency	Percentage
Gender	Male	339	45.69%
	Female	403	54.31%
Age	Younger than 22	64	8.62%
	23 to 30 years old	257	34.64%
	31 to 40 years old	241	32.48%
	41 to 50 years old	140	18.87%
	older than 51	40	23.18%
Education	Less than high school	41	5.79%
	College	75	10.11%
	Undergraduate	435	58.63%
	Master	134	18.06%
	Doctoral degree	55	7.41%
Net monthly income	<2000 yuan	74	9.97%
	2001–4000 yuan	59	7.95%
	4001–6000 yuan	98	13.21%
	6001–8000 yuan	153	20.62%
	8001–12,000 yuan	203	27.36%
	12,001–20,000 yuan	124	16.71%
	>20,001 yuan	31	4.18%
Household size	1 person	38	5.12%
	2 persons	96	12.94%
	3 persons	328	44.2%
	4 persons	161	21.7%
	5 or more persons	119	16.04%
Pregnant female in the family	Yes	10	1.35%
	No	732	98.65%
Older than 60 years	Yes	184	24.8%
	No	558	75.2%

**Table 4:** Ordered logit estimation results of perceptions of contamination of food purchased online by COVID-19 virus and perceiving online food buying as riskier than offline purchases.

Variable Name	Estimated Coefficients			
	(1)	(2)	(3)	(4)
	COVID-19 from online food purchase	COVID-19 from online food purchase	Online purchase riskier than offline purchase	Online purchase riskier than offline purchase
Regional effect	0.359 ***	-	0.198 **	-
	(3.75)		(2.20)	
City effect	-	0.533 ***	-	0.473 ***
		(2.77)		(2.59)
Packaging	0.866 ***	0.869 ***	0.631 ***	0.630 ***
	(9.72)	(9.75)	(7.81)	(7.80)
Social media use	0.314 **	0.338 **	0.320 **	0.323 **
	(2.15)	(2.32)	(2.27)	(2.30)
Gender	0.241 *	0.240*	−0.159	−0.144
	(1.71)	(1.70)	(−1.18)	(−1.06)
Age	0.023	0.010	0.062	0.059
	(0.31)	(0.13)	(0.88)	(0.84)
Education	−0.027	−0.050	−0.035	−0.044
	(−0.31)	(−0.58)	(−0.43)	(−0.54)
Net monthly income	−0.029	−0.035	−0.040	−0.042
	(−0.61)	(−0.76)	(−0.88)	(−0.93)
Household size	0.108	0.118	0.024	0.030
	(1.46)	(1.60)	(0.34)	(0.41)
Pregnant female in the family	−1.176 *	−1.154 *	0.550	0.528
	(−1.72)	(−1.68)	(0.77)	(0.75)
Elderly	−0.254	−0.265	0.099	0.104
	(−1.42)	(−1.48)	(0.57)	(0.61)
N	742	742	742	742
LR chi2(11)	142.78 ***	136.25 ***	82.50 ***	84.42 ***
Pseudo R^2^	0.078	0.075	0.038	0.039

Note: Standard errors in parentheses. *, **, and *** indicate significance of 0.10, 0.05, and 0.01, respectively.

**Table 5:** The mediation effect results of packaging of food purchased online by using the KHB method.

	Regional Effect		City Effect	
	(1)	(2)	(3)	(4)
Dependent variable	COVID-19 from online food purchase	Online purchase riskier than offline purchase	COVID-19 from online food purchase	Online purchase riskier than offline purchase
Total effect	0.448 ***	0.270 ***	0.697 ***	0.603 ***
	(4.69)	(3.01)	(3.63)	(3.32)
Direct effect	0.377 ***	0.218 **	0.561 ***	0.504 ***
	(3.96)	(2.43)	(2.92)	(2.77)
Indirect effect	0.071 *	0.052 *	0.136 *	0.099 *
	(1.85)	(1.83)	(1.75)	(1.74)
Control variables	YES	YES	YES	YES
N	742	742	742	742
Pseudo R^2^	0.08	0.04	0.07	0.04

Note: “YES” means all the control variables were included in the model. To limit the length of the paper, the estimated coefficients of control variables were not listed. *, **, and *** indicate significances of 0.10, 0.05, and 0.01, respectively.

**Table 6:** The mediation effect results of the social media use by respondents.

	Regional Effect		City Effect	
	(1)	(2)	(3)	(4)
Dependent variable	COVID-19 from online food purchase	Online purchase riskier than offline purchase	COVID-19 from online food purchase	Online purchase riskier than offline purchase
Total effect	0.398 ***	0.255 ***	0.601 ***	0.570 ***
	(4.26)	(2.85)	(3.19)	(3.14)
Direct effect	0.379 ***	0.237 ***	0.573 ***	0.545 ***
	(4.04)	(2.63)	(3.04)	(3.00)
Indirect effect	0.019 *	0.018 *	0.028	0.025
	(1.69)	(1.67)	(1.35)	(1.32)
Control variables	YES	YES	YES	YES
N	742	742	742	742
Pseudo R^2^	0.02	0.01	0.02	0.01

Note: “YES” means all the control variables were included in the model. To limit the length of the paper, the estimated coefficients of control variables were not listed. *, and *** indicate significances of 0.10, and 0.01, respectively.

**Table 7:** Robustness test using the replacement model.

	(1) Ordered Probit	(2) OLS	(3) Ordered Probit	(4) OLS
Variable Name	COVID-19 from Online Food Purchase		Online Purchase Riskier than Offline Purchase	
Regional effect	0.208 ***	0.153 ***	0.119 **	0.115 **
	(3.76)	(3.79)	(2.24)	(2.27)
Packaging	0.485 ***	0.346 ***	0.359 ***	0.341 ***
	(9.89)	(10.20)	(7.86)	(8.01)
Social media use	0.193 **	0.151 **	0.171 **	0.169 **
	(2.28)	(2.43)	(2.09)	(2.17)
Control variables	YES	YES	YES	YES
N	742	742	742	742
LRchi2/F	141.95 ***	15.13 ***	80.12 ***	8.39 ***
PseudoR^2^	0.077	0.171	0.037	0.103

Note: “YES” means all the control variables were included in the model. To limit the length of the paper, the estimated coefficients of control variables were not listed. **, and *** indicate significances of 0.05, and 0.01, respectively.

**Table 8:** Heterogeneity analysis: differences between regions/provinces and cities.

Item	(1)	(2)
	COVID-19 from Online Food Purchase	Online Purchase Riskier than Offline Purchase
Regional measure		
Not P province	0	0
	(.)	(.)
P province, but not C city	0.616 ***	−0.0567
	(2.87)	(−0.28)
C city	0.652 ***	0.462 **
	(3.31)	(2.48)
Packaging	0.866 ***	0.630 ***
	(9.73)	(7.81)
Social media use	0.307 **	0.326 **
	(2.10)	(2.31)
Control variables	YES	YES
N	742	742
Loglikelihood ratio	144.59 ***	84.50 ***
Pseudo R^2^	0.079	0.039

Note: “YES” means all the control variables were included in the model. To limit the length of the paper, the estimated coefficients of control variables were not listed. **, and *** indicate significances of 0.05, and 0.01, respectively.

**Table 9:** Consumer risk perception of COVID virus presence in five types of food purchased online (percentage, N = 742).

Food Category	Completely Disagree	Basically Disagree	Neutral	Agree	Completely Agree	Agree and Completely Agree
Online-ordered meal	2.43%	13.88%	38.54%	34.64%	10.51%	45.15%
Fresh agri-products	1.62%	8.89%	26.28%	42.32%	20.89%	63.21%
Vegetables and fruits	2.7%	14.82%	31.4%	35.98%	15.09%	51.07%
Frozen food	1.89%	10.51%	22.91%	39.08%	25.61%	64.69%
Shelf-stable food	5.53%	19.68%	36.12%	29.51%	9.16%	38.67%

Notes: Fresh agri-products include eggs, and fresh aquatic, meat, poultry, and dairy products. Frozen foods include frozen aquatic products, seafood, meat and poultry, and quick-frozen flour foods (dumplings, buns, etc.).

**Table 10:** Heterogeneity analysis of risk perception of infection by coronavirus present on packaging of online purchased food from five groups.

Variable	Online Ordered Meal	Fresh Agri-Products	Vegetables and Fruits	Frozen Food	Shelf-Stable Food
	Marginal Effects (dy/dx)				
Regional effect	0.0186 **	0.0521 ***	0.0117	0.0315*	0.0106
	(2.20)	(3.78)	(1.08)	(1.96)	(1.44)
Packaging	0.100 ***	0.157 ***	0.130 ***	0.178 ***	0.0833 ***
	(8.71)	(11.73)	(10.34)	(12.58)	(7.84)
Social media	−0.000834	0.0408 *	0.0362 **	0.0195	0.0159
	(−0.07)	(1.92)	(2.16)	(0.80)	(1.40)
Control variables	YES	YES	YES	YES	YES
N	742	742	742	742	742

Notes: due to space limitations, the table shows only the marginal effect for the option 5 = completely agree (contains COVID-19 virus). Standard errors in parentheses. Full set of results is available upon request. *, **, and *** indicate significances of 0.10, 0.05, and 0.01, respectively.

## Data Availability

The data used in this study are available upon reasonable request from the authors.

## Appendix A

**Table A1:** Questionnaire.

Gender	Male = 1; Female = 2
Age	Younger than 22 = 1; 23 to 30 = 2; 31 to 40 = 3; 41 to 50 = 4; older than 51 = 5
Education	less than high school = 1; college = 2; undergraduate = 3; Master = 4; Doctoral degree = 5
Net monthly income	Less than 2000 yuan = 1; 2001–4000 yuan = 2; 4001–6000 yuan = 3; 6001–8000 yuan = 4; 8001–12,000 yuan = 5; 12,001–20,000 yuan = 6; more than 20,001 yuan = 7
Household size	1 person = 1; 2 persons = 2; 3 persons = 3; 4 persons = 4; 5 or more = 5
Pregnant female in the family	Yes = 1; No = 2
Older than 60 years	Yes = 1; No = 2
COVIDRi	Respondent location. other regions of non-P province = 1; P province, but not C city = 2; C city = 3
COVIDC	the city with the major epidemic (C city, P province) = 1; other = 0
Packaging	Degree of agreement that online food ingredients or packaging surfaces may contain SARS-COV-2/or its variants: completely disagree = 1; basically disagree = 2; neutral = 3; agree = 4; total agree = 5
Social media use	Do you use online social media to learn about COVID-19? Yes = 1; No = 0
Food purchased online source of COVID-19	Degree of risk of COVID-19 from food purchased online: no risk at all = 1; little risk = 2; neutral = 3; higher = 4; very high risk = 5
Online purchase higher COVID-19 risk	Degree of agreement that online-purchased food is more susceptible to COVID-19 contamination than off-line food: completely disagree = 1; basically disagree = 2; neutral = 3; agree = 4; totally agree = 5
